# Changes to the school food and physical activity environment after guideline implementation in British Columbia, Canada

**DOI:** 10.1186/1479-5868-11-50

**Published:** 2014-04-14

**Authors:** Allison W Watts, Louise C Mâsse, Patti-Jean Naylor

**Affiliations:** 1 School of Population and Public Health, University of British Columbia, F508-4480 Oak Street, Vancouver, British Columbia V6H 3V4, Canada; 2 School of Exercise Science, Physical and Health Education, University of Victoria, PO Box 3015 STN CSC, Victoria, British Columbia V8W 3P1, Canada; 3 Department of Pediatrics, University of British Columbia, F508-4480 Oak Street, Vancouver, BC V6H 3V4, Canada

**Keywords:** School policy, School food availability, Physical education, Implementation

## Abstract

**Background:**

High rates of childhood obesity have generated interest among policy makers to improve the school food environment and increase students’ levels of physical activity. The purpose of this study was to examine school-level changes associated with implementation of the Food and Beverage Sales in Schools (FBSS) and Daily Physical Activity (DPA) guidelines in British Columbia, Canada.

**Methods:**

Elementary and middle/high school principals completed a survey on the school food and physical activity environment in 2007–08 (N = 513) and 2011–12 (N = 490). Hierarchical mixed effects regression was used to examine changes in: 1) availability of food and beverages; 2) minutes per day of Physical Education (PE); 3) delivery method of PE; and 4) school community support. Models controlled for school enrollment and community type, education and income.

**Results:**

After policy implementation was expected, more elementary schools provided access to fruits and vegetables and less to 100% fruit juice. Fewer middle/high schools provided access to sugar-sweetened beverages, French fries, baked goods, salty snacks and chocolate/candy. Schools were more likely to meet 150 min/week of PE for grade 6 students, and offer more minutes of PE per week for grade 8 and 10 students including changes to PE delivery method. School community support for nutrition and physical activity policies increased over time.

**Conclusion:**

Positive changes to the school food environment occurred after schools were expected to implement the FBSS and DPA guidelines. Reported changes to the school environment are encouraging and provide support for guidelines and policies that focus on increasing healthy eating and physical activity in schools.

## Introduction

Over the course of the school day, children have many opportunities to purchase less healthful food and beverages [1-4]. Food and beverages commonly sold at school include soda, pizza, hamburgers, French fries and junk food [1,5,6] and the availability of these foods and beverages has been associated with both greater consumption while at school [3,7] and student’s body weight [8]. Concurrently, children do not achieve adequate levels of physical activity for good health, [9,10] and physical activity while at school has declined over the past several decades [11-13]. Furthermore, children who participate in physical education (PE) classes [14,15] or attend schools that offer daily PE [16] achieve higher levels of physical activity than those who do not participate. Given heightened concern over rising rates of childhood obesity, the school environment has been promoted as a setting where healthy eating and physical activity can be improved [17].

To address population-wide childhood obesity, a growing number of jurisdictions in the U.S., Canada, and abroad have introduced school policies that restrict the types of foods and beverages sold and increase time requirements for physical activity at school. Several studies have found that the implementation of school nutritional policies has restricted the availability [18-21] and consumption [22,23] of less healthful food at school, while others have found little or no association [3,4]. In the US, schools in states with stronger PE laws provided more minutes of PE per week among elementary and middle schools [24,25], but not in high schools [25]. Studies have also found that successful adoption of these policies requires support from members of the school community such as teachers, principals, students and parents [26,27]. Given the importance of reducing childhood obesity and the increase in school policies to address this problem, evaluation of school nutrition and physical activity policies is urgently needed [28].

The purpose of this study was to examine school-level changes associated with the implementation of the Food and Beverage Sales in British Columbia (BC, Canada) Schools (FBSS) and Daily Physical Activity (DPA) guidelines in BC. The FBSS guidelines, initially released in 2005 by the provincial Ministry of Health and Education, provided minimum nutrition standards for food and beverages sold in school vending machines, cafeterias, snack bars as well as sold as part of fundraising activities or at special events [29]. Essentially, the FBSS guidelines restrict what food and beverages can be sold in schools by eliminating high sugar and fat food (e.g. regular chips, candy, pop) entirely and replacing certain food with lower fat, sugar, and salt versions and whole grain substitutes (e.g. whole wheat crust on pizza, baked chips, diet pop) (see FBSS guidelines [29]). In 2007, the FBSS guidelines were revised to align with the Canada’s Food guide [30] and to accelerate implementation of the guidelines by the 2008–09 school year, except for elementary schools where implementation was expected by January of 2008. The DPA guidelines require all schools in BC to offer 30 minutes of daily physical activity in grades K-9 and in grades 10–12 they must document and report 150 minutes per week of physical activity (e.g., through instructional or non-instructional opportunities) (see DPA guidelines [31]). School districts were responsible for developing their own policies and procedures to track implementation of the DPA guidelines [31]. Both guidelines were disseminated through established communication channels, including presentations at meetings, brochures and websites. While schools were expected to implement both the FBSS and DPA guidelines, at the time of this study there were no formal mechanism in place to enforce implementation. The specific aims for this study were to determine whether the school environment changed when the FBSS and DPA guidelines were expected to be fully implemented in the 2008–09 school year. We hypothesized that schools would provide fewer unhealthy options and more healthy options, increase the minutes of PE that students receive per week, change PE delivery from half semester to linear system (i.e., offering PE all versus half the year) and report more stakeholder support for nutrition and physical activity policies.

## Methods

### Sample

In the 2007–08 and 2011–12 school years, all school principals in BC within districts that provided approval were invited to complete a questionnaire on physical activity and nutrition policies and practices at their school. School district approval was received from 43 districts in 2007–08 (73% response rate) and 49 districts in 2011–12 (83% response rate). Independent, Alternate, Francophone and First Nation schools were excluded because this questionnaire was not designed to measure the unique contexts experienced at those schools. In 2007–08, complete surveys were returned from 384 elementary, 118 middle/high and 11 mixed grade schools (48% response rate). Surveys were completed by the principal (90%), vice-principal (7%), teacher (1%) or other staff member (2%). These schools had an average enrollment of 403 students, 60% were located in census metropolitan areas, and schools were from communities with 32% post-secondary education and a median household income of $60,356. In 2011–12, complete surveys were returned from 351 elementary, 125 middle/high and 14 mixed grade schools (49% response rate). Surveys were completed by the principal (93%), vice principal (5%) or a teacher (2%). These schools had an average enrollment of 410 students, 51% were located in census metropolitan areas, and schools were from communities with 32% post-secondary education and a median household income of $58,757. Among this sample, 151 elementary, 50 middle/high and 2 mixed grade schools completed both the 2007–08 and 2011–12 surveys. Note that our first data collection (in 2007–08) occurred before schools were expected to fully implement the FBSS and DPA guidelines in the 2008–09 school year, with the exception of elementary schools which were expected to implement the FBSS guidelines in January of 2008. From our qualitative interviews, we know that schools were still struggling to implement the FBSS guidelines by the 2010–11 school year and that implementation lags over time [26]. While we acknowledge this overlap, it is likely that minimal changes had occurred at our first data collection although some changes could have been initiated.

### Procedures

Upon district approval, school principals were mailed a package that included an invitation letter, a consent form and a hard copy of the questionnaire to be completed and returned using a pre-paid envelope. To increase response rates, schools that had not returned the questionnaire within 4–6 weeks were mailed a second package as well as an email with the option to complete the survey online. Schools also received reminder phone calls by project staff after each mailing. Principals who completed the survey received a $10 gift card. This study was approved by the University of British Columbia and the University of Victoria Research Ethics Board.

### Measures

#### *Food and beverage availability*

Principals completed questions from the School Health Policies and Programs Study (SHPPS) [32] that asked if students had access to the following 11 food and beverage items from the school cafeteria, vending machine or snack bar/store during school hours in a typical week: [1] sugar sweetened beverages (e.g. pop, iced tea, sports drinks or fruit drinks that are not 100% fruit juice); [2] 100% fruit juice; [3] fruit; [4] baked goods (e.g. cookies, crackers, cakes, pastries); [5] low-fat baked goods; [6] French fries [7] salad (lettuce, vegetable, or bean salads), referred to as “vegetables” for this analysis; [8] pizza, hamburgers or hotdogs; [9] salty snacks (e.g. potato chips, cheese puffs); [10] low-fat salty snacks (e.g. pretzels, baked chips); and [11] chocolate candy. Each category was coded as 1 = available; 0 = not available. In 2007–08 principals were given a list and told to check all that apply. In 2011–12, principals were asked to respond (yes/no) on the availability of each item. Additional items were also added to assess if pizzas were served on a whole wheat crust with low-fat cheese and if hotdogs/hamburgers were served on a whole wheat bun with low-fat meat based on the new guidelines for these food items.

#### *Minutes of physical education*

Minutes of PE per week received by grade 6 students was measured as a dichotomous variable. In 2007–08, principals received two versions of this item, the first version (n = 331) provided response categories of: 30–59, 60–89, 90–119, 120–149, >150 minutes per week; while the second version (n = 182) asked the principals to write in the number of PE minutes students in grade 6 received per week. In 2011–12, principals reported the number of days that grade 6 students received PE, the duration of each PE class and if PE was delivered on a semester system. In all versions, minutes of PE per week was dichotomized where 0 = <150 minutes per week and 1 = ≥150 minutes per week to correspond with the BC guidelines.

Minutes of PE per week received by grade 8 and 10 students was measured as a continuous variable. In 2007–08 principals were asked to fill in the minutes per week that grade 8 and 10 students received, separately. In 2011–12, minutes of PE was calculated by multiplying the reported number of days that students received PE by the reported length in minutes of each PE class for grades 8 and 10 separately. If principals reported that PE was delivered on a semester system, the total was divided by 2 to approximate PE received over the entire school year.

#### *Delivery format of physical education*

The delivery method of PE for grade 8 and 10 students was assessed with a dichotomous variable: 0 = delivered in one semester; 1 = PE delivered linearly. In 2007–08, principals were phoned to confirm the delivery method of PE to grade 8 and 10 students. In 2011–12, an item for each grade asked whether PE was required all or part of the year with response options of: not applicable, all year, part of the school year (i.e. 1 semester), or not scheduled.

#### *Stakeholder support*

Principal, staff, parent and student support for nutrition policies was assessed with 4-items, e.g. “Staff support the implementation of policies that increase healthy eating opportunities at school.” Similarly, 4 items assessed support for physical activity guidelines by each stakeholder group, e.g. “Staff support the implementation of policies which increase physical activity for students.” General social pressure to provide healthier food and beverage items and increase physical activity at schools was assessed with two items. All items were measured on a 4-point likert scale (strongly agree to strongly disagree).

#### *Implementation*

The extent of implementation of the food and beverage guidelines in a variety of locations (snack bar, vending machine, cafeteria, fundraising activities, and school events) was assessed in 2011–12. For each location, principals reported the percentage of guideline implementation: 0%, 25%, 50%, 75%, 90%, and 100%. The same question was asked about the extent of implementation of the DPA guidelines for each grade separately. A response of 100% was considered to be full implementation, 25% and 90% was considered to be in process, and 0% was considered to be no implementation.

#### *Socio-demographic variables*

School size was assessed as a continuous variable using the number of students enrolled in each school obtained from the BC Ministry of Education. Community type, the percentage of the population with a high school diploma and median household income were obtained from the 2006 Canadian census and linked to our school-level data using postal codes. Community type was grouped into three categories based on how the census classifies geographical areas: an urban setting that consists of metropolitan areas (population ≥ 100,000 and an urban core ≥ 50,000), a suburban setting (census tract or non-tract agglomeration where urban core ≥10,000), and a rural setting (any area that does not meet criteria for an urban or suburban setting).

### Analysis

Means, standard deviations and percentages were calculated to describe our sample characteristics. Multilevel mixed effects linear and logistic regression was used to model changes over time in availability of food and beverages, minutes per week of PE, delivery method of PE, and school stakeholder support for nutrition and PE policies. Mixed effects modeling was used to account for the clustering of schools within districts and to address the unbalanced nature of our dataset (i.e., schools that contributed data at one or both time points were included in the analysis). This procedure was selected as it is slightly more powerful than examining only schools that provided data in both 2007–08 and 2011–12 (cohort sample).

Models examining changes in the school food environment were conducted separately for elementary and middle/high schools; therefore, mixed grade schools were excluded from the analysis (n = 25). Separate analyses were appropriate because Canadian elementary and middle/high schools differ substantially with respect to their food environment e.g., most elementary schools do not have vending machines or cafeterias. Separate models were also run for each food and beverage item. If less than 10% of schools reported availability of a particular food/beverage the model was not run. To examine changes in PE minutes per week and delivery method, separate models were run for grade 6, 8 and 10 students. All models controlled for school enrollment and community type, education and income. Unadjusted and adjusted odds ratios and 95% confidence intervals were reported for logistic models, while unstandardized and standardized beta coefficients and p-values were reported for linear models. A p-value of less than 0.05 was considered statistically significant. Analyses were conducted using the XTMIXED and XTMELOGIT commands in STATA v.11 (StataCorp, Texas, US).

In sub-analyses, we examined whether the cohort schools differed significantly from schools that participated in 2007–08 only. As compared to cohort schools, those that did not agree to participate again in 2011–12 had similar demographic and socio-economic characteristics, but a smaller proportion of middle/high schools offered French fries (32.4% vs. 54%, p < .001). No other differences were identified. We also conducted analyses in cohort schools only and found that the estimates (e.g., means and percentages) remained unchanged (data not shown). Furthermore, the stability of these estimates was confirmed by testing interactions between time and sample membership (i.e., cohort vs. participated in 2007–08 or 2011–12 only) and no interactions were significant (data not shown) providing further support to combine the two groups in our mixed effects analyses.

## Results

### Implementation

According to middle/high school principals in 2011–12, 66% reported full implementation of guidelines for vending machines, followed by 45%, 36%, 10% and 8% for snack bars, cafeterias, fundraising activities and special events, respectively. Among elementary schools, 22% and 15% reported full implementation of the guidelines for fundraising activities and special events, respectively. The majority of elementary schools did not have a vending machine (78%), cafeteria (95%) or snack bar (88%); therefore, implementation of these guidelines is not presented. For grade 6 students, 65.2% of schools reported full implementation of the DPA guidelines followed by 56% and 51% for grade 8 and 10 students, respectively.

### School nutrition environment

Changes in school availability of specific food and beverages are described in Table 1. Few elementary schools (<10%) reported availability of sugar-sweetened beverages, baked goods, French fries, chocolate & candy, or salty snacks (low-fat and regular) at either time point, thus changes in these items were not modeled. Results from mixed effects modeling reveal that elementary schools had higher odds of having fruit (OR = 2.13, 95% CI = 1.36-3.35) and vegetables (OR = 2.87, 95% CI = 1.51-5.44) and lower odds of having 100% fruit juice (OR = 0.40, 95% CI = 0.25-0.65) available in the 2011–12 school year (Table 2). There was no change in the availability of pizza, hamburgers & hotdogs or low-fat baked goods in elementary schools. Middle/high schools had lower odds of having sugar-sweetened beverages (OR = 0.51, 95% CI = 0.30-0.88), regular baked goods (OR = 0.28, 95% CI = 0.15-0.52), chocolates & candy (OR = 0.06, 95% CI = 0.01-0.36), regular salty snacks (OR = 0.11, 95% CI = 0.05-0.25) and French fries (OR = 0.30, 95% CI = 0.11-0.80) available in the 2011–12 school year. No other food or beverage products significantly changed over time. In the 2011–12 school year, after the guidelines were expected to be implemented, most principals reported that their school was serving pizza, hamburgers and hotdogs with whole wheat crust or buns: 59%, 72% and 69%, respectively, among elementary schools and 56%, 70% and 62%, respectively, among middle/high schools.

**Table 1:** Description of the school nutrition and physical education environment of schools, British Columbia, Canada

	**Elementary Schools**		**Middle/High Schools**	
	**2007–08 (n = 384)**	**2011–12 (n = 351)**	**2007–08 (n = 118)**	**2011–12 (n = 125)**
	**Mean (SD)/%**			
**Availability of Food and Beverages**				
Sugar-sweetened Beverages	5.5%	5.7%	62.7%	44.8%
French Fries	0	0.3%	41.5%	25.6%
Chocolate and Candy	0	0	37.3%	10.4%
Regular Salty Snacks	0.3%	1.14%	40.7%	7.2%
Regular Baked Goods	2.9%	4.3%	53.4%	26.4%
Low-fat Salty Snacks	6.3%	8.0%	78.8%	75.2%
Low-fat Baked Goods	10.4%	13.7%	70.3%	74.4%
Pizza, Hotdogs, Hamburgers	13.3%	18.0%	63.6%	68.0%
Fruit	28.9%	39.3%	74.6%	78.4%
100% Fruit Juice	49.0%	34.5%	95.8%	92.8%
Vegetables	9.4%	17.4%	69.5%	66.4%
**Physical Education**				
Grade 6			-	-
< 150 min PE/week	65.9%	51.9%		
≥150 min PE/week	34.1%	48.1%		
Grade 8	-	-		
PE minutes/week			180.1 (30.4)	199.1 (47.1)
Linear vs. Semester	-	-	67.0%	79.0%
Grade 10	-	-		
PE minutes/week			184.0 (23.7)	194.0 (38.2)
Linear vs. Semester	-	-	50.5%	39.8%
**Support for Nutrition Policies**				
General Social pressure	2.73 (0.70)	2.71 (0.66)	2.74 (0.68)	2.62 (0.66)
Principal Support	3.49 (0.52)	3.34 (0.51)	3.39 (0.54)	3.29 (0.49)
Staff Support	2.77 (0.58)	3.16 (0.44)	2.67 (0.66)	3.06 (0.45)
Student Support	2.57 (0.61)	2.70 (0.63)	2.42 (0.74)	2.44 (0.65)
Parent Support	2.79 (0.63)	2.92 (0.53)	2.77 (0.78)	3.0 (0.59)
**Support for Physical Education Policies**				
General Social Pressure	2.43 (0.69)	2.64 (0.68)	2.18 (0.69)	2.33 (0.58)
Principal Support	2.57 (0.66)	2.70 (0.65)	2.57 (0.66)	2.70 (0.65)
Staff Support	2.89 (0.60)	3.15 (0.48)	2.29 (0.67)	2.73 (0.60)
Student Support	3.34 (0.57)	3.21 (0.48)	2.50 (0.69)	2.66 (0.36)
Parent Support	2.40 (0.63)	3.11 (0.47)	2.14 (0.69)	2.79 (0.52)

**Table 2:** Changes in availability after implementation of school food and beverage guidelines, British Columbia, Canada (2007/08–2011/12)

	**Elementary Schools**				**Middle/High Schools**			
	**Unadjusted models**		**Adjusted models**^**1**^		**Unadjusted models**		**Adjusted models**^**1**^	
	**N**	**OR (95% CI)**	**N**	**OR (95% CI)**	**N**	**OR (95% CI)**	**N**	**OR (95% CI)**
Sugar-sweetened Beverages		-		-	238	**0.52** (0.31-0.87)	236	**0.51** (0.30-0.88)
French Fries		-		-	241	**0.26**(0.09-0.76)	238	**0.30** (0.11-0.80)
Chocolate and Candy		-		-	240	**0.07** (0.01-0.40)	238	**0.06** (0.01-0.36)
Regular Salty Snacks		-		-	239	**0.10** (0.04-0.24)	237	**0.11** (0.05-0.25)
Regular Baked Goods		-		-	240	**0.26** (0.14-0.49)	238	**0.28** (0.15-0.52)
Low-fat Salty Snacks		-		-	239	0.87 (0.41-1.88)	237	1.03 (0.53-2.01)
Low-fat Baked Goods	715	1.51 (0.79-2.89)	707	1.54 (0.81-2.91)	240	1.51 (0.79-2.89)	237	1.48 (0.79-2.79)
Pizza, Hotdogs, Hamburgers	712	1.70 (0.96-2.89)	704	1.65 (0.95-2.88)	239	1.46(0.76-2.8)	236	1.47 (0.80-2.71)
Fruit	715	**1.98** (1.25-3.15)	707	**2.13** (1.36-3.35)	239	1.71 (0.75-3.91)	237	1.67 (0.79-3.53)
100% Fruit Juice	718	**0.40** (0.25-0.65)	710	**0.40** (0.25-0.65)	241	0.58 (0.10-3.44)	238	0.64 (0.12-3.34)
Vegetables	716	**2.84** (1.49-5.42)	708	**2.87** (1.51-5.44)	240	0.96 (0.41-2.25)	238	1.02 (0.48-2.18)

^1^Mixed effects logistic regression models were adjusted for neighborhood-level median household income, percent with post-sec education, school size, and school location.

OR, odds ratio; CI, confidence interval.

Bolded odds ratios are statistically significant at p < 0.05.

### Physical education

Changes to the duration and delivery method of PE are described by grade in Table 1. After adjusting for school and community variables, schools had higher odds of meeting the recommended amount of 150 minutes per week of PE for grade 6 students (OR = 2.01, 95% CI = 1.00-4.3) and provided more minutes of PE to grade 8 (b = 17.72, p < .001) and grade 10 students (b = 11.17, p = 0.01) in 2012 (Table 3). Schools had higher odds of providing PE linearly to grade 8 students and in a semester format for grade 10 students in 2011–12 as compared to 2007–08.

**Table 3:** Changes in physical education after implementation of the physical activity guidelines, British Columbia, Canada (2007/08–2011/12)

		**Unadjusted models**			**Adjusted models**^**a**^		
		**N**	**Odds Ratio (95% CI)**	**b (p-value)**	**N**	**Odds Ratio (95% CI)**	**b (p-value)**
Grade 6	PE (≥150 min/week)^b^	401	2.10 (0.97-4.44)	--	388	**2.01** (1.00-4.30)	--
Grade 8	PE min/week^c^	227	--	**18.66** (<.001)	218	--	**17.72** (<.001)
	Delivery (Linear)^d^	231	**3.77** (1.10-12.92)	--	222	**3.45** (1.15-10.28)	--
Grade 10	PE min/week^b^	199	--	**9.12** (0.04)	192	--	**11.17** (0.01)
	Delivery (Linear)^d^	204	0.51 (0.19-1.36)	--	197	0.42 (0.15-1.19)	--

^a^Models were adjusted for neighborhood-level median household income, percent with post-sec education, school size, and school location.

^b^Mixed effect logistic regression examining the odds of offering greater than or equal to 150 minutes of PE per week versus offering less than 150 minutes per week, in 2011–12 as compared to 2007–08.

^c^Mixed effect linear regression, mean increase in minutes of PE per week between 2007–08 and 2011–12.

^d^Mixed effect logistic regression examining the odds of offering PE as a linear course versus a semester course in 2011–12 as compared to 2007–08.

CI, confidence interval; b, unstandardized regression coefficient; Bolded estimates are statistically significant at p < 0.05.

### School community support

Levels of stakeholder support are presented in Table 1. After controlling for school and community variables, principal reported staff and parent support for healthy eating and physical activity policies significantly increased in elementary and middle/high schools (Table 4). Student support for healthy eating policies increased while principal support decreased among elementary schools, with no changes among middle/high schools. Student support for physical activity policies decreased among elementary schools but increased among middle/high schools as did social pressure to provide more physical activity at school. Principal support for physical activity policies did not change over time.

**Table 4:** **Changes in stakeholder support after implementation of school guidelines, British Columbia, Canada (2007/08–2011/12)**^**a,b**^

	**Elementary Schools**			**Middle/High Schools**		
	**N**	**b**	**p-value**	**N**	**b**	**p-value**
**School Nutrition**						
Social Pressure	703	-0.30	.55	231	-0.10	.20
Principal Support	712	**-0.14**	<.001	235	-0.98	.14
Staff Support	704	0.40	<.001	231	**0.39**	<.001
Student Support	575	**0.15**	.004	226	0.02	.82
Parent Support	710	**0.11**	.02	233	**0.24**	.003
**Physical Education**						
Social Pressure	704	**0.20**	<.001	230	**0.18**	.02
Principal Support	702	-0.05	.27	222	0.14	.06
Staff Support	699	**0.26**	<.001	222	**0.43**	<.001
Student Support	709	**-0.11**	.003	222	**0.19**	.02
Parent Support	705	**0.69**	<.001	238	**0.07**	<.001

^a^Separate models were run for each support variable.

^b^Mixed effects linear models were adjusted for neighborhood-level median household income, percent with post-sec education, school size, and school location.

b, unstandardized regression coefficient.

Bolded estimates are statistically significant at p < 0.05.

## Discussion

This study is one of very few that examined the impact of both nutrition and physical activity school guidelines on the school environment. Results demonstrate that after BC schools were expected to implement the food and beverage guidelines and daily physical activity requirements, schools decreased their availability of less healthful food and beverages and increased the number of minutes of PE offered per week, based on principal report. These changes are encouraging and similar to the policy impacts found in other jurisdiction [18-21]. Further evaluation to determine if these policies will impact student behaviors is warranted.

### Change in school nutrition environment

The FBSS guidelines resulted in significant changes to the school food environment but impacted elementary and middle/high schools differently. Differences may be partly explained by the amount of food and beverages available at school to start with [4,20]. Overall, middle/high schools decreased the availability of less healthful food and beverages whereas elementary schools decreased the availability of less healthful beverages and significantly increased the availability of fruits and vegetables at school. The increase in fruits and vegetables was somewhat unexpected since the guidelines did not mandate schools to offer more healthy options but may be the result of the government scaling up provincial programs that targeted elementary schools over this time period such as the fruit and vegetable program that included bi-weekly delivery, raising awareness of local produce, ‘tasting’ activities, and materials for teachers, and a whole school physical activity and healthy eating initiative that provided technical support and resources to teachers that were attempting to implement classroom healthy eating practices [33].

Overall, nutritional environment changes in elementary and middle/high schools were all in the expected direction. With the exception of the availability of pizza, hamburgers and hotdogs and availability of 100% fruit juices (only in middle/high schools), the guidelines seem to have targeted the appropriate food and beverage items. It is not surprising that the availability of pizza, hamburgers and hotdogs did not change over time because the guidelines allowed reformulation of these products as opposed to restriction. Reformulations focused on improving grains and reducing fat, sodium and additives rather than increasing vegetables. Indeed, we found that after implementation of the guidelines a large proportion of schools (>50%) were serving these items with whole grains. If children continue to eat only these reformulations, the guidelines might fall short in increasing children’s consumption of vegetables at lunch time. Furthermore, elementary schools significantly decreased the availability of 100% fruit juice while middle and high schools significantly decreased the availability of sugar-sweetened beverages but not 100% fruit juices where availability remained high (94%). Although 100% fruit juice was not targeted by the guidelines, it may have been used to replace sugar-sweetened beverages in middle/high school vending machines. When consumed in appropriate portions, 100% fruit juice is not problematic; [34] however, large servings of 100% fruit juice have been suggested as a potential contributor to children’s positive energy balance [35,36]. Continued monitoring of such policies is essential to ensure that consumption of sugar-sweetened beverages is not replaced by consumption of inappropriate portions of 100% fruit juice.

Many schools reported that they continue to struggle with making changes to their food environment. Implementation was particularly low for fundraising activities and special events among both elementary and middle/high schools as well as for cafeterias among middle/high schools. As we discovered from in-depth interviews with school principals and teachers in 2010–11, fundraising activities and special events may be particularly challenging areas for schools to make changes since access to healthier alternatives are lacking and there is a potential for lost revenue [26]. In addition, school informants reported that perceived value, compatibility with school mandate/teaching philosophy, observable positive impacts, and availability of resources promoted implementation while the complexity of guidelines impeded implementation. Previous studies have also reported that schools experience a variety of barriers to implementation including funding, competing priorities and support from stakeholders [27]. Furthermore, a Canadian study identified lack of support, complexity of guidelines, and a top-down approach as barriers to implementation [37]. A key area of future research needs to be in improving implementation as gaps in implementation likely result in reduced policy effectiveness.

Previous studies have also reported favorable changes to the school food environment after implementation of state or federal policies/guidelines [19,21,38]. A cross-sectional examination of the strength of state nutrition policies found that elementary and middle schools with state policies restricting junk food had lower availability of junk food in vending machines and school stores, with no change in high schools [19]. This result differs from the current study, where we found declines in less healthful food among middle/high schools but not elementary schools. In another study, records of all food served for lunch were collected for the years before and after implementation of the Texas nutrition policy and revealed that fewer fried vegetables (French fries) were served for both elementary and high schools, but no change was observed for regular vegetables, fruit or milk [38]. Using a similar design as the current study, comparison before and after the implementation of Maine’s state-wide nutrition policy banning “foods of minimal nutritional value” found that the availability of soda, but not other junk foods, declined in high schools [21]. In contrast, we observed declines in various less healthful food products including junks food, along with declines in sugar-sweetened beverages. Despite the encouraging changes within the BC school food environment, many schools continued to serve refined grains and other less healthful food and beverages.

### Change in school physical activity environment

After the DPA policy was put into place, schools increased minutes of PE per week for grade 6, 8, and 10 students. Unlike most US state policies, the BC guidelines were not specific about increasing PE time but instead allowed schools choice in how to increase physical activity. In elementary schools, schools could meet the guidelines by implementing the Action Schools! BC initiative that was supported by the province. A key strategy of Action Schools! BC was to increase physical activity breaks in the classroom [39,40]. While the DPA guidelines did not directly target PE time, we found that many schools opted to increase PE time to meet the provincial guidelines. This finding is corroborated by results from our qualitative study that examined (among others) strategies schools used to implement the DPA guidelines [26]. Our data did not allow for comparison of the impact of the two approaches in achieving the guideline. In the province of Ontario, where a similar DPA policy was mandated in 2005, a cross-sectional study using accelerometry reported that 5-years into implementation of the policy, the majority of students were not active on every school day and no child met sustained activity for 20 minutes at one time, suggesting that schools were not meeting the guidelines [41]. Further investigation is required to investigate the relative value of a DPA guideline versus a daily PE guideline.

In the current study, schools also changed their delivery method of PE from semester to linear for grade 8 students, while the opposite occurred for grade 10 students. This is a puzzling finding. We had hypothesized that schools might move towards a linear system to deliver PE (shorter bouts spread out over the whole year); however, this finding aligns with our qualitative interviews that suggest schools are challenged with fitting activity into a very busy school schedule [26]. This did not seem to be the case for students in grade 10. Unlike in grades 6 and 8, PE in grade 10 is a graduation requirement. Perhaps more grade 10 schools opted to increase physical activity classes and as a result offered PE on a semester system which is easier to accommodate in the schedule given that all other academic courses are on a semester system.

Although the guidelines examined in the current study were not specific to PE, several US studies have reported similar positive changes to PE time after implementation of PE-specific policies [24,25]. A study of elementary schools found that schools were more likely to meet the recommended 150 minutes per week of PE once the state policy was in place [24]. In another study of middle/high schools, minutes of PE per week increased by 18 minutes for grade 8 students and 11 minutes for grade 10 students. Increased minutes of PE time also occurred after policy implementation in US high schools [25]. These increases are meaningful as a recent study found that students did not compensate by being less active at home; thus, school PE increased their overall physical activity levels [42].

### Change in school community support for healthier guidelines

The successful changes made by schools in this study occurred concurrently with increased support from the school community for healthier eating and physical activity. Greater support by school community has previously been identified as key component to successful implementation of school policies/guidelines [26,27,43]. Increases in staff and parent support were consistent across school and guideline type suggesting that support from these two groups is important for fostering change. In general, support increased more for physical activity policies than healthy eating policies. This may be a consequence of the differing implementation context. Changes in DPA are largely the responsibility of the teacher and have no impact on fundraising while changes in food policies involve parents and affect fundraising. In addition, the lowest increase in support for healthy eating was among middle/high school students, where support was also lowest at baseline. Restrictions on food may be particularly unpopular among this age group as they are developing independence and have access to choices off school property when schools do not provide them [26,44]. There was no change in policy support by the principal; however, they also had self-reported the highest level of initial support for both healthy eating and physical activity across school types.

### Limitations

Although this study focused on the implementation of guidelines within the BC context, similar policies are being adopted in other jurisdictions making these findings relevant to other settings. This study was limited to mostly principal reports of the school environment which may be influenced by social desirability bias, unmeasured characteristics of the respondent, or differences in respondents at each time point. However, our self-report showed some validity as associations between implementation and school environment were in the expected direction (higher implementation significantly associated with less unhealthy food and beverages and more PE minutes - data not shown). While we acknowledge the limitations of self-report, studies that are able to utilize objective measures such as food purchasing data or direct observation of school cafeterias may find different results. Importantly, some changes were made as to how the questions were asked at each time point; this specifically pertains to the PE questions (see the Methods section). Every effort was made to treat data in a way that enhanced comparability over time but it is possible that changing the questions influenced the results as principals may have interpreted the question differently. Our analysis incorporated cross-sectional samples at each time point which may have contributed to bias in our findings. This impact is likely minimal since our results are stable with and without the inclusion of the cross-sectional samples (see Methods section). Furthermore, an important limitation of natural experiments is that we cannot control for other concurrent school, community or provincial programs that may contribute to observed changes in the school environment. As there were provincial initiatives targeted at elementary schools during our evaluation period, the changes observed in elementary schools may have been attributed to both the enactment of the FBSS or DPA guidelines and support provided for these initiatives. Finally, we did not examine changes in student level behaviors; however, this will be an important component of future policy evaluations, particularly in light of evidence that suggests increases in PE time may results in decreased forms of other school-based physical activity, such as at recess, [24] and that students may turn to food and beverages available off school property if they can’t get them at school [26,44]. Despite these limitations, this study capitalized on a natural experiment with data collected before and after full implementation of guidelines were expected, providing a rare opportunity to demonstrate policy impact at the school-level.

## Conclusions

Positive changes to the school food environment occurred after schools were expected to implement the FBSS and DPA guidelines. Fewer middle/high schools offered unhealthy food and more elementary schools offered healthy food. Simultaneously schools were providing more minutes of PE to students. Our findings also identified several key areas that might require more attention in future evaluation. Previous research has linked changes in the school environment with student’s nutrition and physical activity behaviors and obesity, [1,8,16] thus the changes observed are encouraging and highlight the relevance of policy interventions to enhancing the health environment in schools.

## Competing interests

The authors declare that they have no competing interests.

## Authors’ contributions

AW conducted the data analysis and contributed to interpreting the results and writing and editing the manuscript. LM contributed to conceptualizing the study, interpreting the results, and writing and editing the manuscript. PJN contributed to conceptualizing the study, interpreting the results, and editing the manuscript. All authors have read and approved the final manuscript.
